# Transporters in vitamin uptake and cellular metabolism: impacts on health and disease

**DOI:** 10.1093/lifemeta/loaf008

**Published:** 2025-03-10

**Authors:** Yaxuan Yuan, Ligong Chen

**Affiliations:** State Key Laboratory of Membrane Biology, School of Pharmaceutical Sciences, Key Laboratory of Bioorganic Phosphorus Chemistry and Chemical Biology (Ministry of Education), Tsinghua University, Beijing 100084, China; State Key Laboratory of Metabolic Dysregulation & Prevention and Treatment of Esophageal Cancer, Innovation Center of Basic Research for Metabolic-Associated Fatty Liver Disease, Ministry of Education of China, Tianjian Laboratory of Advanced Biomedical Sciences, Academy of Medical sciences, Zhengzhou University, Zhengzhou, Henan, China, 450001; State Key Laboratory of Membrane Biology, School of Pharmaceutical Sciences, Key Laboratory of Bioorganic Phosphorus Chemistry and Chemical Biology (Ministry of Education), Tsinghua University, Beijing 100084, China; State Key Laboratory of Metabolic Dysregulation & Prevention and Treatment of Esophageal Cancer, Innovation Center of Basic Research for Metabolic-Associated Fatty Liver Disease, Ministry of Education of China, Tianjian Laboratory of Advanced Biomedical Sciences, Academy of Medical sciences, Zhengzhou University, Zhengzhou, Henan, China, 450001

**Keywords:** vitamin, vitamin deficiencies, ADME, SLC proteins, drug targets

## Abstract

Vitamins are vital nutrients essential for metabolism, functioning as coenzymes, antioxidants, and regulators of gene expression. Their absorption and metabolism rely on specialized transport proteins that ensure bioavailability and cellular utilization. Water-soluble vitamins, including B-complex and vitamin C, are transported by solute carrier (SLC) family proteins and ATP-binding cassette (ABC) transporters for efficient uptake and cellular distribution. Fat-soluble vitamins (A, D, E, and K) rely on lipid-mediated pathways through proteins like scavenger receptor class B type I (SR-BI), CD36, and Niemann-Pick C1-like 1 (NPC1L1), integrating their absorption with lipid metabolism. Defective vitamin transporters are associated with diverse metabolic disorders, including neurological, hematological, and mitochondrial diseases. Advances in structural and functional studies of vitamin transporters highlight their tissue-specific roles and regulatory mechanisms, shedding light on their impact on health and disease. This review emphasizes the significance of vitamin transporters and their potential as therapeutic targets for deficiencies and related chronic conditions.

## Introduction

Vitamins are a diverse group of organic compounds indispensable for human health, required in trace amounts from dietary sources. While they do not supply energy, they play crucial roles in cellular functions and biochemical processes essential for sustaining life. The concept of vitamins emerged in the late 19th century, with Polish biochemist Casimir Funk coining the term “vitamin” in 1912 by combining “vita” (life) and “amine”, reflecting the initial identification of thiamine [1, 2].

Throughout human evolution, the capacity to synthesize most vitamins has been lost, making dietary intake essential [3]. Although some vitamins, such as vitamins D, K_2_, B_1_, B_2_, B_3_ and B_7_, are synthesized in small amounts through endogenous pathways or by the gut microbiota [4–6]—for example, vitamin B_3_ from tryptophan (Trp) or vitamin D through ultraviolet (UV) radiation exposure—these processes do not produce sufficient quantities to meet daily requirements. Consequently, dietary sources remain critical for maintaining optimal health.

Vitamins are categorized based on their solubility into two primary groups: fat-soluble vitamins (A, D, E, and K) and water-soluble vitamins (B-complex and C) [7]. Table 1 provides an overview of key vitamins, including their chemical structures, dietary sources, recommended daily allowances (RDAs), and biological functions, serving as a framework for understanding their physiological importance. Fat-soluble vitamins primarily function as hormones, antioxidants, or regulators of gene transcription, while water-soluble vitamins predominantly act as coenzymes or enzymatic precursors. This classification underscores their diverse physiological roles and highlights the distinct ways in which the body processes these vitamins.

**Table 1.** Name, structure, sources, recommended daily allowances (RDAs), and biological functions of vitamins.

Vitamin name	Structure of derivatives	Source and RDAs (adults)	Biological roles
A (retinol)	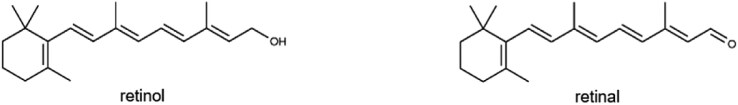	Carrots, sweet potatoes, spinach, liver, and eggs Male: 900 µg Female: 800 µg	Cellular repair, immune function, antioxidant, fetal development, and vision
D (cholecalciferol)	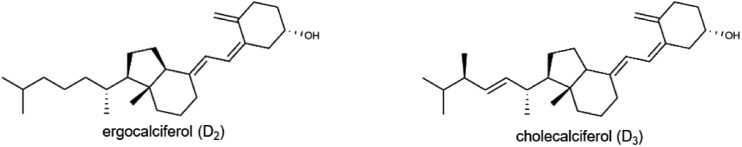	Fatty fish (salmon, mackerel, and tuna), fortified dairy products, sun exposure 15 µg (600 IU)	Bone and dental mineralization, calcium and phosphorus absorption, metabolism, and immune modulation
E (α-tocopherol)	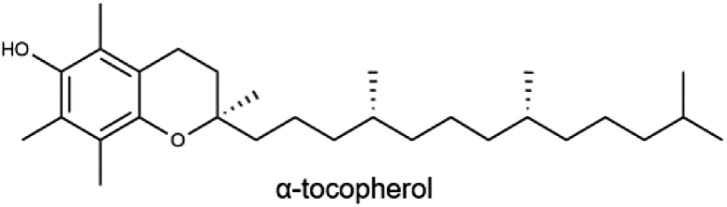	Nuts, wheat germ, vegetable oils (corn and sunflower), spinach, and fortified cereals 15 mg	Antioxidant, protecting cells and unsaturated fatty acids, heme synthesis
K (phylloquinone)	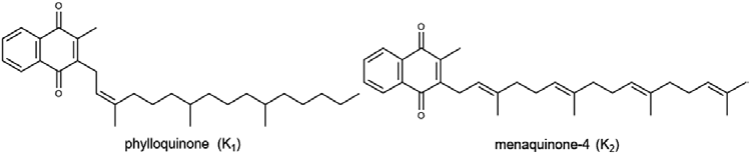	Leafy greens (kale and spinach), broccoli, and soybeans Male: 120 µg Female: 90 µg	Blood clotting, synthesis of prothrombin and clotting factors (VII, IX, X), bone metabolism, participating in protein carboxylation processes
B_1_ (thiamine)	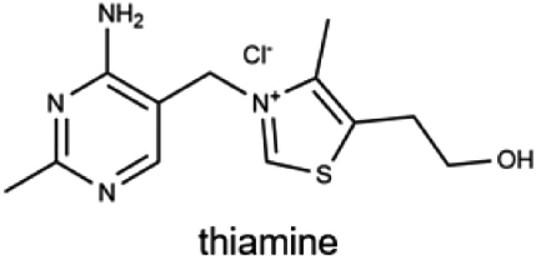	Whole grains, pork, legumes, liver, and nuts Male: 1.2 mg Female: 1.1 mg	Energy metabolism, coenzyme in decarboxylation (pyruvate and α-keto acid), and neuronal function
B_2_ (riboflavin)	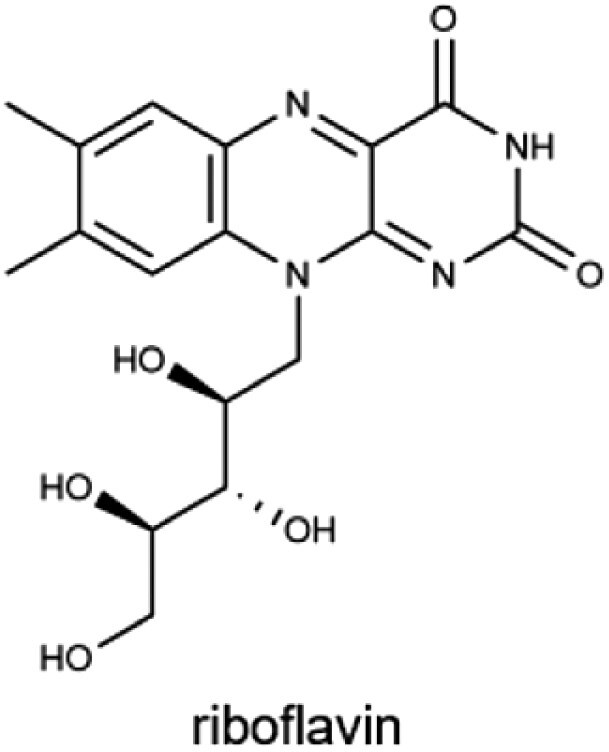	Dairy products, eggs, lean meats, and green vegetables Male: 1.3 mg Female: 1.1 mg	Energy metabolism, component of FAD and FMN in electron transport, and supporting mucosal and ocular health
B_3_ (niacin)	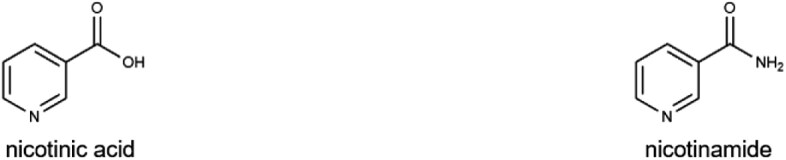	Poultry, fish, fortified cereals, and peanuts Male: 16 mg Female: 14 mg	Coenzymes NAD, NADP for hydrogen transfer, sex hormone production, and glycogen synthesis
B_5_ (pantothenic acid)	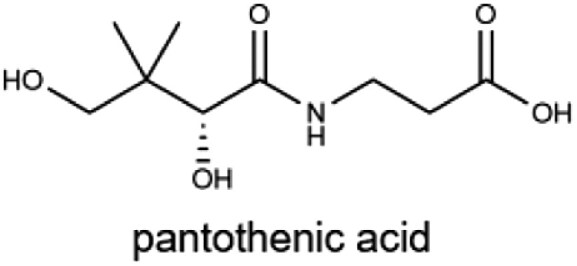	Chicken, beef, whole grains, and avocados 5 mg	Energy metabolism, antibody and corticosteroid synthesis, and coenzyme in transamination for synthesis of amino acids
B_6_ (pyridoxine)	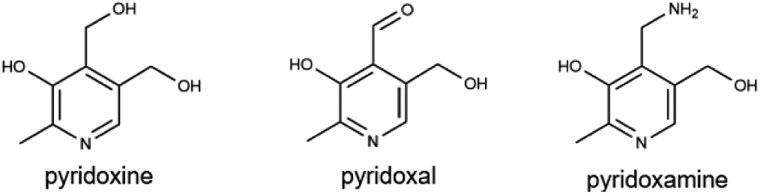	Poultry, fish, potatoes, bananas, and fortified cereals Male: 1.3−1.7 mg Female: 1.3−1.5 mg	Fat, protein metabolism, coenzyme in transamination, hemoglobin production, and neuronal function
B_7_ (biotin)	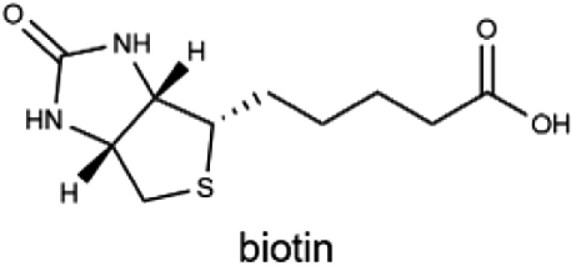	Eggs, nuts, whole grains, and salmon 30 µg	Fatty acid and glucose synthesis, coenzymes in carboxylation reactions, and genetic functions
B_9_ (folate)	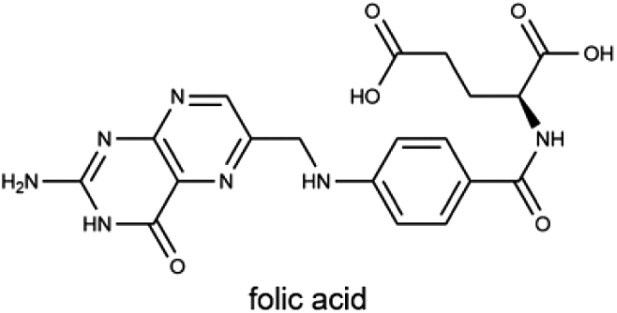	Leafy greens, citrus fruits, legumes, and fortified grains 400 µg Pregnancy: 600 µg*	Cell growth and division, coenzyme in DNA/RNA synthesis, and supporting leukocyte and erythrocyte formation
B_12_ (cobalamin)	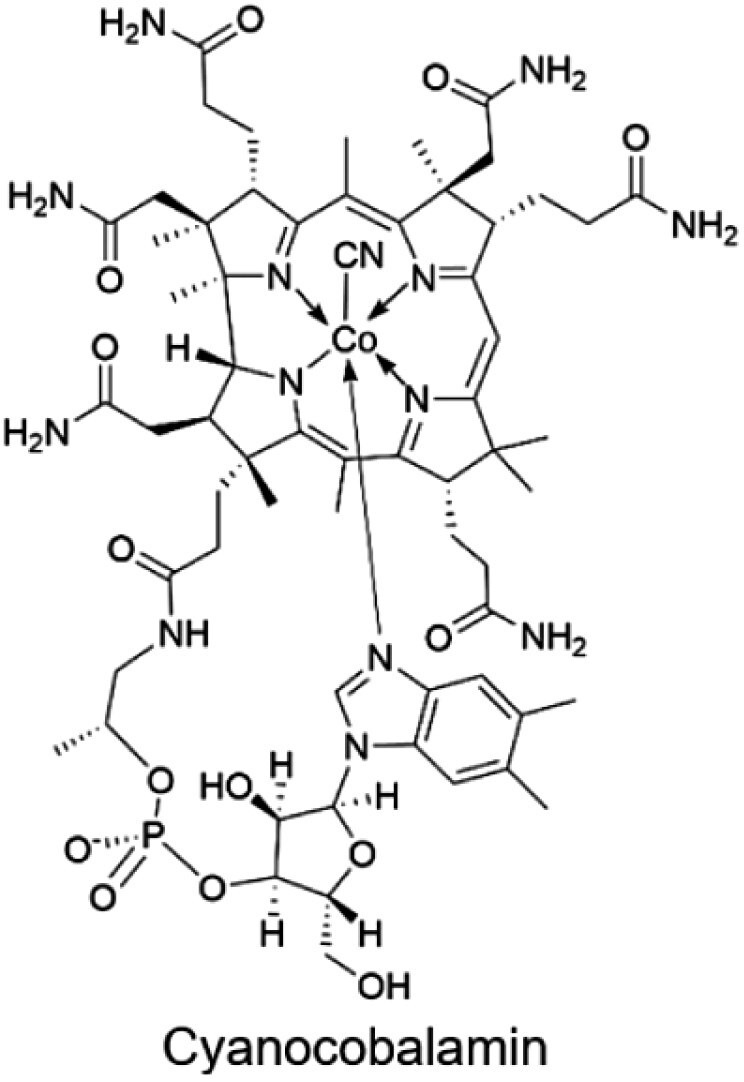	Animal products (meat, eggs, poultry, fish, and dairy) and fortified cereals 2.4 µg	Hematopoiesis, erythrocyte cell maturation, DNA/RNA synthesis, lipid and protein metabolism, coenzyme in reduction of ribonucleotides to deoxyribonucleotides, iron absorption, neuronal function, and aiding in myelin synthesis
C (ascorbic acid)	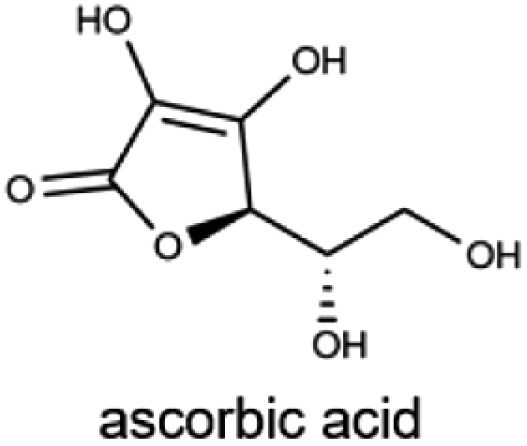	Citrus fruits, strawberries, bell peppers, and broccoli Male: 90 mg Female: 75 mg	Collagen synthesis, iron absorption, wound healing, supporting connective tissue repair, and antioxidant

RDAs: recommended daily allowances, represents the average daily dietary intake level sufficient to meet the nutrient requirements of healthy individuals. RDA values in this table are primarily based on the Dietary Reference Intakes (DRIs) established by the U.S. National Academies of Sciences, Engineering, and Medicine (NASEM, https://ods.od.nih.gov).

The absorption, distribution, metabolism, and excretion (ADME) characteristics [8] of these two groups differ markedly, influencing their storage, toxicity, and deficiency risks. Fat-soluble vitamins require dietary fats and bile for absorption and enter the bloodstream via the lymphatic system [9]. Their storage in the liver and adipose tissue minimizes deficiency risks but increases toxicity potential when consumed in excess [10]. Conversely, water-soluble vitamins, except for B_12_, are absorbed through specific transport proteins in the small intestine and are rapidly excreted by the kidneys [11]. Due to their limited storage, consistent dietary intake is necessary to avoid deficiencies. These properties associated with the ADME framework are summarized in Table 2.

**Table 2.** Vitamin classification based on ADME properties.

	Fat-soluble vitamins	Water-soluble vitamins
**Sources**	Diet (except vitamin D, synthesized via ultraviolet exposure) Gut bacteria synthesize small amounts of vitamin K	Primarily from diet Gut bacteria synthesize small amounts of vitamins B_7_, B_9_, and B_12_
**Absorption**	Depends on intestinal and pancreatic function Requires the formation of dietary lipids, bile acids, and micelles	Requires specific luminal transporters in the intestine Sodium- or proton-coupled transport systems are essential for uptake
**Distribution**	Transported via chylomicrons and lipoproteins (LDL and HDL) Stored long-term in liver and adipose tissue	Circulates freely in plasma or binds to carrier proteins Minimal storage (exceptions: B_9_ in liver for 3–4 months; B_12_ for 3–4 years)
**Metabolism**	• Vitamin A → retinoic acid and retinal • Vitamin D → 1α,25-dihydroxyvitamin D • Vitamin E → tocopherol and tocotrienol • Vitamin K → hydroquinone Function as hormones, antioxidants, or regulators of gene transcription	Converted into coenzymes or active derivatives • Vitamin B_1_ → thiamine pyrophosphate (TPP) • Vitamin B_2_ → FMN and FAD • Vitamin B_3_ → NAD^+^ and NADP^+^ • Vitamin B_6_ → pyridoxal phosphate (PLP) • Vitamin B_9_ → tetrahydrofolate (THF)
**Excretion**	Slow elimination, increasing accumulation risks	Rapid renal excretion, minimizing accumulation risks
**Vitamin deficiency**	Caused by malabsorption (e.g. celiac disease, cystic fibrosis), bile acid deficiency, or medication interference • Vitamin A: night blindness, dry skin, and xeropthalmia • Vitamin D: rickets (children) and osteomalacia (adults) • Vitamin E: erythrocyte hemolysis and nerve damage • Vitamin K: excessive bleeding and coagulation disorders	Caused by restricted diets (e.g. veganism), or malabsorption disorders (e.g. gastritis, gastric resection) • Vitamin B_1_: beriberi and Wernicke encephalopathy • Vitamin B_3_: pellagra (dermatitis, diarrhea, and dementia) • Vitamin B_12_: anemia and neuropathy • Vitamin C: scurvy and bleeding gums
**Vitamin toxicity**	More frequent toxicity due to storage • Vitamin A: headache, dizziness, liver damage, and birth defects • Vitamin D: hypercalcemia and calcium deposition	Rare due to efficient clearance • Vitamin B_6_: peripheral neuropathy and skin lesions • Vitamins B_1_, B_2_, B_5_, B_7_, B_9_, and B_12_: no toxicity

The absorption and distribution of vitamins rely on specific transport proteins, including primary active transporters, secondary active transporters, and facilitated transporters [12]. Primary active transporters, such as the ATP-binding cassette (ABC) transporter family, harness ATP hydrolysis to translocate substrates across membranes [13]. Secondary active transporters utilize the electrochemical potential generated by transmembrane ion gradients to transport substrates against concentration gradients. In contrast, facilitated transporters mediate the passive movement of substrates along the concentration gradients without requiring energy input. Additionally, studies on vitamin transport have identified key vitamin receptors that trigger intracellular signaling cascades through structural rearrangements, facilitating vitamin endocytosis [14].

The investigation of vitamin transport proteins bridges nutritional science and oncology. In nutritional research, the focus centers on the transport mechanisms in the intestines and kidneys, which are vital for the physiological roles of vitamins and are directly linked to deficiency-related symptoms. Epidemiological studies have revealed how variations in vitamin transporter expression among populations influence the prevalence of specific diseases, such as hematological disorders and developmental abnormalities. In oncology, research highlights the therapeutic potential of vitamin transport proteins. Strategies targeting these proteins aim to regulate their expression or function, thereby enhancing cancer treatment efficacy and minimizing adverse effects on healthy tissues. The solute carrier (SLC) family has recently emerged as a key area of interest due to its essential role in vitamin absorption and distribution [15]. Overexpression of SLC proteins in tumor cells offers a promising target for developing innovative drug delivery systems.

The causes of vitamin deficiencies are diverse, including dietary habits, physiological conditions, medication effects, and genetic factors. Modern dietary patterns, which often feature high-energy processed foods with reduced micronutrient content, contribute significantly to these deficiencies. For example, the widespread adoption of Western diets, typically low in essential vitamins, exacerbates nutrient gaps. Industrial food processing, such as milling and germ removal, further depletes thiamine levels [16]. Additionally, vegan diets can lead to vitamin B_12_ deficiencies due to the lack of animal-derived sources [17–20]. These dietary trends, coupled with physiological changes during pregnancy and aging, highlight a growing public health challenge, as vitamin deficiencies continue to drive global health issues, particularly lifestyle-related diseases [3, 21, 22]. Certain medications, including anticonvulsants and antituberculosis drugs, can interfere with vitamin transport and metabolism, leading to deficiencies. Genetic mutations may also disrupt the function of vitamin transport proteins, contributing to the development of deficiencies. Furthermore, the interdependence of vitamins often results in the simultaneous occurrence of multiple deficiencies.

## Individual vitamins: from molecular function, transport mechanisms to pathological significance

### Vitamin B_1_

Vitamin B_1_, or thiamine, plays a critical role in energy production, nerve function, and various metabolic processes. It exists in several forms—free thiamine, thiamine monophosphate (TMP), thiamine pyrophosphate (TPP), and thiamine triphosphate (TTP)—each serving distinct functions across different tissues [23]. Despite its presence in a wide range of foods, many populations worldwide remain at risk of clinical or subclinical thiamine deficiency. In low- and middle-income countries, the limited prevalence of thiamine fortification, combined with dietary monotony and reliance on refined grains as staple foods, often leads to deficiencies [16, 24]. These deficiencies can cause severe health conditions such as beriberi and polyneuritis, with children and adolescents being particularly vulnerable. Even in regions where overt symptoms like beriberi are uncommon, subclinical thiamine deficiencies have been associated with impaired neurocognitive development. This underscores the importance of monitoring thiamine levels globally, even in areas where severe manifestations are rare [16].

#### Absorption and transport of vitamin B_1_

Vitamin B_1_ absorption occurs in the small intestine through active and passive mechanisms, depending on dietary intake levels. Phosphorylated dietary thiamine undergoes dephosphorylation by intestinal phosphatases, releasing free thiamine for absorption. At low intake levels (< 5 mg) [25], active transport is mediated by thiamine transporters THTR1 and THTR2, encoded by the *SLC19A2* and *SLC19A3* genes, respectively [26–28]. THTR1 operates at both the apical and basolateral membranes of enterocytes, while THTR2 is restricted to the apical membrane [29, 30]. Non-specific organic cation transporters (OCT1 and OCT3, encoded by *SLC22A1* and *SLC22A3* genes, respectively) also facilitate thiamine transport through cation-proton exchange [31]. Additionally, the reduced folate carrier (RFC1, encoded by *SLC19A1*) primarily mediates the transport of TMP as an anion exchanger. Thiamine and TPP produced by gut microbiota can also be absorbed in the colon via the high-affinity transporter, the colonic TPP transporter (cTPPT) encoded by the *SLC44A4* gene, contributing further to thiamine availability [32, 33].

#### Distribution and metabolism of vitamin B_1_

After absorption, THTR1 promotes the entry of thiamine into the portal circulation, while the transport to tissues involves THTR1, THTR2, and OCT transporters [30]. In the bloodstream, thiamine is primarily non-specifically bound to albumin. Within cells, free thiamine is phosphorylated by thiamine pyrophosphate kinase (TPK1) to form TPP, the biologically active coenzyme [34]. Approximately 80% of cellular thiamine exists as TPP, with mitochondria utilizing 90% of this pool for oxidative phosphorylation and energy production [35]. TPP also plays a critical role in cytoplasmic transketolase reactions and is transported into mitochondria via a high-affinity transporter encoded by the *SLC25A19* gene [36].

#### Impact of genetic mutations of vitamin B_1_ transport protein

Genetic mutations in thiamine transport proteins can impair thiamine metabolism, leading to a range of associated diseases. Mutations in the *SLC19A2* gene cause thiamine-responsive megaloblastic anemia (TRMA), a condition characterized by megaloblastic anemia, non-autoimmune diabetes, and early-onset sensorineural deafness [37, 38]. Although THTR2 and passive transport mechanisms can partially compensate for THTR1 dysfunction, THTR1 remains the sole thiamine transporter in bone marrow, pancreatic β-cells, and cochlear cells, making its dysfunction particularly debilitating [39]. Variants in the *SLC19A3* gene are associated with Wernicke-Korsakoff syndrome [40] and biotin-thiamine-responsive basal ganglia disease [41, 42], both of which respond to high-dose thiamine supplementation [35]. Furthermore, the *SLC44A4* gene has been linked to ulcerative colitis [43], emphasizing the broader physiological importance of thiamine transport systems.

Thiamine transport proteins also interact with various drugs, influencing nutritional status and therapeutic outcomes [44]. Prolonged use of diuretics such as furosemide increases urinary thiamine excretion, often resulting in deficiency [45]. Metformin, a common treatment for type 2 diabetes, affects THTR2 function, thereby altering thiamine absorption and metabolism. Fedratinib, a Janus kinase 2 inhibitor used to treat myelofibrosis, exhibits off-target inhibitory effects on *SLC19A2* and *SLC19A3*, further underscoring the clinical relevance of these transport proteins [46–48].

#### Recent advances in the transport of vitamin B_1_

Recent advances in structural biology have enhanced our understanding of thiamine transport [49]. Cryo-electron microscopy (Cryo-EM) studies of SLC19A3 in complex with thiamine, vitamin B_6_, or Fedratinib have provided valuable insights into transporter–drug interactions, improving our comprehension of thiamine transport mechanisms and informing drug design strategies [50]. Additionally, SLC35F3, a newly identified thiamine transporter, has been linked to plasma thiamine levels and hypertension. Mutations in *SLC35F3* gene may reduce transport efficiency, highlighting its potential as a therapeutic target for conditions like hypertension [51, 52]. These findings underscore emerging opportunities to further explore thiamine metabolism and its broader implications for health and disease.

### Vitamin B_2_

Vitamin B_2_, or riboflavin (Rf), is essential for cellular redox reactions, facilitating electron transfer between two-electron donors (e.g. NAD(H) and succinate) and one-electron acceptors (e.g. iron-sulfur and cytochrome proteins) [53]. Rf serves as a precursor for flavin mononucleotide (FMN) and flavin adenine dinucleotide (FAD), which are essential for flavoproteins involved in key metabolic processes [54], including mitochondrial oxidative phosphorylation, fatty acid β-oxidation, and antioxidant defense [54]. Although Rf-rich foods, such as dairy, eggs, and leafy greens, are widely available [55], certain populations or regions remain at risk for inadequate intake [56]. For example, over 5% of Australian adults report insufficient Rf intake, with prevalence increasing with age [57]. Vitamin B_2_ deficiency impairs flavoprotein function, leading to clinical manifestations, such as glossitis, dermatitis, neuromuscular dysfunction, and an increased susceptibility to systemic diseases like anemia and neurodegeneration.

#### Absorption and transport of vitamin B_2_

Vitamin B_2_ absorption occurs primarily at the brush border of the small intestine through specific Rf transporters (RFTs), including RFVT1 (*SLC52A1*) [58], RFVT2 (*SLC52A2*) [59], and RFVT3 (*SLC52A3*) [60]. RFVT3 is the primary transporter responsible for intestinal Rf uptake, a process influenced by pH levels. In contrast, RFVT1 and RFVT2 predominantly mediate Rf transport from the basolateral side into the portal circulation [61]. The expression of RFTs varies across tissues: RFVT1 is mainly expressed in the intestinal epithelium, placenta, and skin, where it facilitates active Rf transport; RFVT2 shows high expression in the nervous system with lower levels in other tissues; RFVT3 is widely distributed across various tissues, including the small intestine and prostate [61].

#### Distribution and metabolism of vitamin B_2_

In the bloodstream, Rf is transported to cell membranes via Rf-binding proteins (RfBP) and subsequently taken up by cells through RFTs [62]. Inside cells, Rf undergoes enzymatic conversion to FMN and FAD via ATP-dependent flavokinase (FRK) and FAD synthetase (FADS) [63]. These flavin derivatives are vital cofactors for over 90 flavoproteins, including 75 that require FAD, 9 that depend on FMN, and 6 that serve as Rf transporters or cytosolic enzymes.

During intracellular metabolism, SLC25A17 facilitates the transport of FAD and FMN into peroxisomes, maintaining redox balance and providing antioxidant protection [64]. SLC25A32 ensures mitochondrial FAD availability for oxidative phosphorylation [65]. Notably, SLC25A17 also mediates the transport of coenzyme A (CoA) and nicotinamide adenine dinucleotide (NAD^+^), underscoring its multifunctional role in peroxisomal metabolism.

#### Impact of genetic mutations of vitamin B_2_ transport protein

Defects in Rf transport proteins are strongly linked to various severe clinical conditions, particularly neuromuscular and metabolic disorders [66]. Mutations in the *SLC52A2* and *SLC52A3* genes impair Rf transport, leading to flavoprotein dysfunction and neuromuscular diseases, such as Brown-Vialetto-Van Laere syndrome (BVVL), which is characterized by optic atrophy and muscle weakness [67]. Similarly, heterozygous mutations in *SLC52A1* can cause transient symptoms of multiple acyl-CoA dehydrogenase deficiency (MADD) in newborns, especially when maternal Rf levels are insufficient during pregnancy [68]. Mutations in the *SLC25A32* gene disrupt FAD availability, impairing cellular energy metabolism and producing symptoms that range from mild exercise intolerance to severe ataxia [65]. Rf supplementation has been shown to increase intracellular FAD levels, compensating for transport deficiencies and restoring enzyme activity.

#### Recent advances in the transport of vitamin B_2_

Rf transport proteins exhibit tissue-specific roles that are essential for health. Retbindin (Rtbdn), a retina-specific Rf-binding protein, is crucial for maintaining high Rf levels in the retina [69]. Positioned between photoreceptor cells and the retinal pigment epithelium, Rtbdn helps sustain optimal Rf concentrations necessary for retinal health. Rtbdn deficiency markedly depletes retinal Rf, exacerbating photoreceptor degeneration in inherited retinal diseases [70]. Additionally, SLC22A14, an Rf transporter expressed specifically in sperm, plays a key role in supporting sperm function and fertility [71]. Deficiency in SLC22A14 disrupts sperm metabolism and can result in male infertility [71]. Research on SLC22A14 not only enhances our understanding of male reproductive biology but also highlights opportunities to develop novel contraceptives. These findings underscore the diverse roles of Rf transport proteins in maintaining health. Moreover, they suggest potential strategies for preventing or treating related diseases through targeted regulation of specific transport proteins.

### Vitamin B_3_

Vitamin B_3_, or niacin (NA), exists primarily in two forms: NA and nicotinamide (NAM). Both act as precursors for NAD⁺ and its phosphorylated form, nicotinamide adenine dinucleotide phosphate (NADP⁺). These coenzymes are essential for redox reactions, energy metabolism, and cellular repair [72, 73]. NAD⁺ is synthesized through three main pathways: (i) the *de novo* kynurenine pathway (from Trp), (ii) the Preiss-Handler pathway (from NA), and (iii) the salvage pathway (from NAM, nicotinamide riboside [NR], or nicotinamide mononucleotide [NMN]) [74, 75]. Trp thus also serves as an indirect dietary source of vitamin B_3_. Severe vitamin B_3_ deficiency results in pellagra [76, 77], which is marked by the “three Ds”: dermatitis, diarrhea, and dementia. Subclinical deficiencies, however, increase the risk of anemia, neurodegeneration, and cardiovascular diseases [78]. Pellagra is particularly common in regions where maize constitutes a major part of the diet. To combat this, many countries have implemented food fortification programs with NA [79].

#### Absorption and transport of vitamin B_3_

Vitamin B_3_ absorption occurs primarily in the small intestine, facilitated by specific transport proteins. Sodium-coupled monocarboxylate transporter 1 (SMCT1), encoded by *SLC5A8*, serves as the primary high-affinity NA transporter in the terminal jejunum and ileum. SMCT2, encoded by *SLC5A12*, also functions in the jejunum, though with lower affinity [80, 81]. In the colon, monocarboxylate transporter 1 (MCT1), encoded by *SLC16A1*, plays a more prominent role, particularly under elevated NA concentrations [82]. NAM is primarily absorbed through passive diffusion. Hepatic NA transport is mediated by organic anion transporter 2 (OAT2, encoded by *SLC22A7*), which facilitates NAD⁺ production and metabolic regulation [83]. In the kidneys, OAT10 (encoded by *SLC22A13*) is responsible for the renal reabsorption of NA and its derivatives [84].

#### Distribution and metabolism of vitamin B_3_

After absorption, vitamin B_3_ is distributed systemically and converted intracellularly to fulfill its physiological functions. In the Preiss-Handler pathway, NA enters cells through specific transporters, including SLC5A8, SLC5A12, SLC16A1, SLC22A13, and SLC22A7. Within the cell, NA undergoes a three-step enzymatic conversion to NAD⁺, catalyzed by NA phosphoribosyltransferase (NAPRT), NMN adenylyltransferases (NMNAT), and NAD⁺ synthase (NADS) [85]. For *de novo* NAD⁺ synthesis, Trp is subsequently converted into quinolinic acid (QA) through intermediates such as kynurenine and 3-hydroxyanthranilic acid. QA is then metabolized into nicotinic acid mononucleotide (NAMN) by quinolinate phosphoribosyltransferase (QPRT), ultimately contributing to NAD⁺ biosynthesis.

The salvage pathway is the primary mechanism for NAD⁺ biosynthesis in mammalian cells, recycling NAM produced during NAD⁺ consumption by enzymes such as sirtuin deacetylases (SIRT1–7), poly(ADP-ribose) polymerases (PARP1–2), cADP-ribose synthases (CD38 and CD157), and the NAD⁺ glycohydrolase SARM1 (Sterile alpha and Toll/interleukin receptor [TIR] motif-containing protein 1) [86–89]. NAM is either methylated by nicotinamide N-methyltransferase (NNMT) for urinary excretion or converted back into NMN by extracellular enzymes CD38 and CD157 [90, 91]. The NMN transporter SLC12A8 [92], recently identified, facilitates NMN uptake, while NMN is also converted to NR by CD73 [93]. NR enters cells via equilibrative nucleoside transporters (ENTs), encoded by *SLC29A1*−*4*.

Subcellular compartmentalization plays a crucial role in regulating NAD⁺ availability [74]. The mitochondrial transporter SLC25A51 [94, 95] and the liver-specific mitochondrial transporter SLC25A47 facilitate NAD⁺ import into mitochondria, supporting oxidative phosphorylation [96]. Similarly, the peroxisomal transporter SLC25A17 delivers NAD⁺ into peroxisomes for β-oxidation [64, 97]. However, the mechanisms governing NAD⁺ transport into the Golgi apparatus remain unknown.

#### Impact of genetic mutations of vitamin B_3_ transport protein

Mutations in vitamin B_3_ transport proteins disrupt NA metabolism and availability, contributing to metabolic disorders. For instance, mutations in *SLC5A8* impair nicotinic acid absorption, potentially causing pellagra-like symptoms even with adequate dietary intake. Similarly, mutations in *SLC22A13* reduce renal NA reabsorption, leading to NAD⁺ depletion, oxidative stress, and mitochondrial dysfunction. Variants in *SLC22A13* have also been associated with increased risks of neurodegenerative diseases, including Alzheimer’s disease (AD) and Parkinson’s disease. Additionally, genetic alterations in *SLC22A7* compromise hepatic NAD⁺ metabolism, potentially exacerbating cardiovascular and metabolic conditions [83].

#### Recent advances in the transport of vitamin B_3_

Recent studies have identified critical mechanisms underlying NA transport and its therapeutic potential. The discovery of SLC12A8 as a specific NMN transporter highlights the importance of the salvage pathway in maintaining intracellular NAD⁺ levels, particularly during metabolic stress, aging, or disease [92]. This pathway presents promising therapeutic targets to counteract NAD⁺ depletion. Beyond serving as an NAD⁺ precursor, NA exerts metabolic effects through its interaction with the G-protein-coupled receptor GPR109A [98]. At pharmacological doses, NA binds to GPR109A in adipose tissue, inhibiting lipolysis and decreasing free fatty acid release [99]. This action reduces plasma levels of very-low-density lipoprotein (VLDL) and low-density lipoprotein (LDL), establishing NA as a valuable therapeutic option for cardiovascular disease management [100, 101]. Furthermore, NA has demonstrated benefits in improving muscle strength and alleviating fatty liver in patients with mitochondrial myopathy [102]. While NA may cause side effects, it remains an effective and viable treatment for hyperlipidemia.

### Vitamin B_5_

Vitamin B_5_, or pantothenic acid (Pan), serves as a critical precursor for CoA and acyl carrier protein (ACP), both of which are essential for carbohydrate, fat, and protein metabolism [103]. CoA is vital for cellular acetylation reactions, while ACP plays a central role in fatty acid synthesis. Vitamin B_5_ deficiency is extremely rare due to its widespread availability in food sources and endogenous production by gut microbiota [30]. However, deficiencies have been reported in specific scenarios, such as in malnourished individuals during World War Ⅱ or controlled human trials. These cases resulted in “burning feet syndrome”, characterized by neuropathy and severe extremity pain [103]. Additionally, cerebral vitamin B_5_ deficiency has been associated with neurodegeneration and dementia, including AD [104].

#### Absorption and transport of vitamin B_5_

Dietary vitamin B_5_ primarily exists as CoA or ACP, which requires enzymatic hydrolysis to release free Pan for absorption. Pan is absorbed mainly in the small intestine through both active and passive mechanisms. At low concentrations, absorption occurs via the sodium-dependent multivitamin transporter (SMVT), encoded by *SLC5A6*, located on the apical membrane of intestinal epithelial cells [105–107]. SMVT also mediates the absorption of biotin and lipoic acid, a vitamin-like antioxidant. This process is sodium-dependent, driven by the electrochemical gradient of Na^+^ across the membrane, where SMVT functions as a sodium-coupled symporter for its substrates [108]. At higher concentrations, Pan can also enter cells through passive diffusion. Once absorbed, Pan is excreted primarily as unmetabolized Pan in urine. At physiological levels, most Pan is reabsorbed in the kidney tubules, ensuring minimal loss [103].

#### Distribution and metabolism of vitamin B_5_

After absorption, Pan is distributed systemically and passively taken up by red blood cells. However, red blood cells cannot convert Pan into CoA due to their lack of mitochondria. High-metabolic-demand tissues, such as the liver, kidneys, adrenal glands, and heart, actively transport Pan through the SMVT for intracellular use. Once inside cells, Pan undergoes a tightly regulated multistep process to synthesize CoA [109, 110]:

(i) Phosphorylation: Pantothenate kinase (PANK) catalyzes this ATP-dependent step, converting Pan into 4′-phosphopantothenate (PPan). This rate-limiting step is regulated by feedback inhibition from CoA derivatives.
(ii) Cysteine addition: Phosphopantothenoylcysteine synthetase (PPCS) adds cysteine to form phosphopantothenoylcysteine (PPanC).
(iii) Decarboxylation: Phosphopantothenoylcysteine decarboxylase (PPC-DC) converts PPanC into 4′-phosphopantetheine (PPanSH).
(iv) Mitochondrial transport: The mitochondrial transporter SLC25A42 imports PPanSH into mitochondria.
(v) CoA formation: Coenzyme A synthase (COASY) completes CoA synthesis via ATP-dependent adenylation and dephosphorylation.

CoA and its acyl derivatives are regulated by compartmentalization and inter-organelle fluxes [109]. In the endoplasmic reticulum (ER), acetyl-CoA is imported via the transporter SLC33A1 (AT-1) in exchange for CoA, a mechanism essential for protein and glycan acetylation [111–114]. In peroxisomes, ABC transporters, such as ABCD1−3, import acyl-CoA molecules into the peroxisomal matrix to support β-oxidation and other metabolic functions. Additionally, SLC25A17 actively transports CoA into peroxisomes [64], ensuring its availability for critical enzymatic reactions.

#### Impact of genetic mutations of vitamin B_5_ transport protein

Mutations in vitamin B_5_ transport proteins disrupt CoA metabolism, resulting in severe metabolic disorders. Homozygous mutations in *SLC25A42*, which mediates CoA transport into the mitochondria in exchange for nucleotides [115], have been associated with lactic acidosis, mitochondrial myopathy, developmental regression, and seizures. While Pan supplementation can restore CoA levels in fibroblasts with *SLC25A42* mutations, clinical outcomes remain limited, underscoring the complexity of compensatory metabolic pathways [116]. Similarly, dysregulation of SLC33A1 has been linked to developmental and degenerative diseases, highlighting its critical role in protein acetylation and autophagy [112, 113, 117].

#### Recent advances in the transport of vitamin B_5_

Vitamin B_5_ exhibits therapeutic potential in several contexts. It has demonstrated efficacy in treating corneal thinning [118, 119] and alleviating rheumatoid arthritis symptoms [120], effects attributed to its anti-inflammatory and antioxidant properties [121]. Pan is also essential for maintaining CoA homeostasis, with supplementation showing promise in mitigating cellular defects in TANGO2 (transport and Golgi organization 2 homolog) deficiency, as evidenced in *Drosophila* and human cell models [122]. The biosynthesis of CoA is tightly regulated by enzymes such as PANK, whose dysfunction is linked to neurodegeneration with brain iron accumulation (NBIA) [123–126]. Mutations in the *PPCS* gene, encoding the enzyme responsible for the second step of CoA biosynthesis, have been associated with autosomal recessive dilated cardiomyopathy [127]. Despite significant advances, research on CoA transport within the Golgi apparatus remains limited. Understanding its role in glycosylation and protein modification could uncover novel therapeutic strategies for metabolic and neurodegenerative disorders.

### Vitamin B_6_

Vitamin B_6_ encompasses a group of chemically related compounds, including pyridoxal (PL), pyridoxamine (PM), and pyridoxine (PN), along with their phosphorylated derivatives: pyridoxal 5′-phosphate (PLP), pyridoxamine 5′-phosphate (PMP), and pyridoxine 5′-phosphate (PNP) [128]. Of these, PLP is the biologically active form of vitamin B_6_ and serves as a coenzyme in over 160 enzymatic reactions. Additionally, PLP functions as an antioxidant, helping to alleviate oxidative stress [129]. While deficiencies are rare, they can occur in conditions, such as chronic alcoholism, diabetes, celiac disease, and as a side effect of certain medications. These deficiencies can lead to neurological symptoms, including seizures and depression.

#### Absorption and transport of vitamin B_6_

In the intestine, dietary vitamin B_6_ is primarily absorbed in its dephosphorylated forms (PL, PM, and PN) after hydrolysis by intestinal phosphatases [130]. The absorption process involves passive diffusion at high concentrations and carrier-mediated mechanisms at physiological concentrations. Notably, the thiamine transporters THTR1 and THTR2, traditionally known for thiamine uptake, also mediate the transport of various vitamin B_6_ forms [50, 131].

Once inside the enterocytes, PL, PN, and PM are phosphorylated by pyridoxal kinase (PLK) to PLP, PNP, and PMP, respectively. Pyridoxamine 5′-phosphate oxidase (PNPO), an FMN-dependent enzyme, then oxidizes PNP and PMP to PLP through the PLP salvage pathway [132, 133]. *In vitro* studies using human intestinal epithelial Caco-2 cells revealed that, upon incubation with different forms of vitamin B_6_, only PL was detected on the basolateral side. This finding suggests that other forms of vitamin B_6_ are converted into PL within the intestine [134].

#### Distribution and metabolism of vitamin B_6_

After absorption, vitamin B_6_ is transported via the portal vein to the liver, where PLK phosphorylates it to PLP. In the bloodstream, PLP binds to albumin for transport to various tissues. Since PLP cannot directly cross cell membranes, it is either released as an albumin-bound complex or dephosphorylated by extracellular tissue-nonspecific alkaline phosphatase (TNSALP) into PL, which can then enter cells and cross the blood-brain barrier [135]. To prevent toxicity, PLP levels are tightly regulated by pyridoxal phosphatase (PDXP). PLP functions as a coenzyme in numerous pathways, including neurotransmitter synthesis (e.g. serotonin, dopamine, and gamma-aminobutyric acid [GABA]), amino acid metabolism, notably through alanine aminotransferase-mediated transamination pivotal to gluconeogenesis, and carbohydrate metabolism via glycogen phosphorylase in glycogenolysis. Additionally, PLP supports nucleic acid synthesis, vitamin cofactor activation (vitamins B_12_ and B_2_), and lipid metabolic processes [128, 136].

#### Impact of genetic mutations of vitamin B_6_ transport protein

Genetic mutations in *SLC19A2* and *SLC19A3* can impair the cellular uptake of vitamin B_6_, leading to neurological and hematological disorders. These transporters are primarily known for thiamine uptake, but their involvement in vitamin B_6_ transport suggests that patients with thiamine-related conditions, such as thiamine-responsive TRMA, may also experience vitamin B_6_ deficiency complications. However, further research is needed to fully understand this relationship.

#### Recent advances in the transport of vitamin B_6_

Recent research has emphasized the therapeutic potential of vitamin B_6_ analogs [137]. For instance, pyridoxine-dependent epilepsy (PDE), caused by *PNPO* mutations, can be effectively managed with PLP or PN supplementation [138, 139]. Similarly, mutations in the *ALDH7A1* (aldehyde dehydrogenase 7A1) gene, which encodes an enzyme involved in amino acid catabolism, have been associated with vitamin B_6_-dependent epilepsy [140, 141]. These mutations lead to the accumulation of toxic byproducts in PLP-dependent reactions, resulting in seizures that often respond to vitamin B_6_ supplementation.

Studies on the dynamic regulation of PLP have highlighted the crucial role of dephosphorylation and rephosphorylation processes in controlling tissue-specific PLP distribution. Hypophosphatasia, a rare genetic disorder caused by mutations in the *TNSALP* gene, illustrates the importance of dephosphorylation mechanisms in PLP bioavailability [142, 143]. Further investigation into novel PLP transporters could reveal new insights into vitamin B_6_ metabolism and provide therapeutic strategies for metabolic and neurological disorders.

### Vitamin B_7_

Vitamin B_7_, or biotin, is a vital cofactor for several biotin-dependent carboxylases involved in key metabolic processes, including gluconeogenesis, fatty acid synthesis, and the catabolism of odd-chain fatty acids and branched-chain amino acids [144]. Additionally, biotin regulates gene expression and modulates intracellular signaling pathways [145, 146]. Its strong affinity for avidin has made the “biotin-avidin system” a widely used tool in research [147].

Biotin deficiency can result in growth retardation, skin abnormalities, and neurological disorders. In animal models, maternal biotin deficiency is directly linked to congenital malformations and increased mortality [148, 149]. Certain populations are at higher risk for biotin deficiency or suboptimal levels, including individuals with inborn errors of biotin metabolism, those on chronic anticonvulsants, alcoholics, pregnant women, and people with inflammatory bowel disease [150].

#### Absorption and transport of vitamin B_7_

Dietary biotin exists in two forms: free biotin and protein-bound biotin. Additionally, gut microbiota primarily synthesize biotin in the colon. Protein-bound biotin is hydrolyzed by gastrointestinal proteases and peptidases into biotin-L-lysine (biocytin) and biotin-oligopeptides. These compounds are further cleaved by biotinidase, releasing free biotin for absorption [151].

Free biotin is primarily absorbed in the small intestine, predominantly through the SMVT [106, 152–154], as discussed in Section “Absorption and transport of vitamin B_5_”. Structural analogs of biotin with a free carboxyl group on the pentanoic acid side chain can inhibit this uptake. Radiolabeled uptake studies reveal that biotin and Pan share similar affinities for SMVT (biotin: 3.2 µmol/L; pantothenate: 1.5 µmol/L). SMVT is widely expressed across various tissues, including the brain, placenta, liver, pancreas, and kidneys [155–157].

Furthermore, Na^+^-dependent carrier-mediated mechanisms have been observed in human colonic epithelial cell models, such as NCM460 cells [158]. Recent studies indicate the presence of a single, saturable transport system in specific cells, including keratinocytes and peripheral blood mononuclear cells (PBMCs) [159, 160]. In PBMCs, the affinity constant for biotin is significantly higher (2.6 ± 0.4 nmol/L), potentially mediated by MCT1, suggesting the involvement of biotin in immune function and inflammation [160].

#### Distribution and metabolism of vitamin B_7_

After biotin enters cells via SMVT, it binds to holocarboxylase synthetase (HCLS), converting inactive precursors into active carboxylases [161]. These biotinylated enzymes catalyze crucial metabolic reactions: (i) Fatty acid synthesis: Acetyl-CoA carboxylase (ACC) converts acetyl-CoA to malonyl-CoA, thereby regulating the balance between fatty acid synthesis and oxidation; (ii) Gluconeogenesis and TCA cycle: Pyruvate carboxylase (PC) converts pyruvate to oxaloacetate, which is essential for maintaining glucose levels and metabolic equilibrium; (iii) Branched-chain amino acid metabolism: Propionyl-CoA carboxylase (PCC) converts propionyl-CoA to methylmalonyl-CoA, which is further metabolized to succinyl-CoA for entry into the TCA cycle; (iv) Leucine metabolism: β-methylcrotonyl-CoA carboxylase (MCC) converts β-methylcrotonyl-CoA to β-methylglutaconyl-CoA, preventing the accumulation of toxic intermediates. In addition to its enzymatic functions, biotin undergoes recycling via biotinidase, forming a dynamic “biotin cycle” that ensures its continuous availability for cellular processes [144]. Biotin also regulates gene expression through incorporation into histones via biotinylation, which modulates chromatin structure and influences transcriptional activity [161].

#### Impact of genetic mutations of vitamin B_7_ transport protein

Mutations in biotin transport proteins can have significant clinical consequences. For example, compound heterozygous mutations in *SLC5A6* are associated with multisystem dysfunction, including developmental delays and metabolic acidosis [162]. High-dose biotin supplementation, combined with Pan and lipoic acid, has been effective in bypassing SMVT defects through passive diffusion, improving metabolic balance [163]. Furthermore, mutations in *SLC5A6* may result in immune deficiencies, as observed in a family with low immunoglobulin levels and keratinization issues. These mutations impair B-cell differentiation and antibody production, but biotin supplementation has been shown to reverse these defects [164]. Recent studies using *SMVT* knockout mouse models revealed that intestinal biotin transport deficiency triggers gut inflammation, mediated by microbiota alterations. This underscores the critical role of SMVT in maintaining biotin homeostasis and supporting immune stability [165].

#### Recent advances in the transport of vitamin B_7_

Biotin supplementation has emerged as a promising therapeutic approach for neurological and dermatological disorders associated with biotin deficiency or metabolic defects, such as biotinidase deficiency, HCLS deficiency, and biotin-thiamine-responsive basal ganglia disease (BTBGD) [155]. For example, high-dose biotin has effectively treated BTBGD, which is linked to defects in THTR2, potentially by stimulating *SLC19A3* expression [162]. Additionally, extremely high biotin doses (100−300 mg/day) have been suggested as a treatment for multiple sclerosis, although the underlying mechanisms remain unclear. Notably, some children with biotin-responsive conditions do not exhibit known biotin deficiencies, highlighting a gap in our understanding of the molecular basis of these cases.

Emerging evidence indicates that biotin uptake extends beyond SMVT, with additional high-affinity transport systems, such as MCT1, identified in specific cell types. Furthermore, recent studies suggest that free carboxyl groups on the pentanoic acid chain of biotin are crucial for SMVT-mediated uptake, while chemical modifications (e.g. amide or ester groups in biotin conjugates) impede transport [154]. This underscores the importance of understanding the transport mechanisms for biotin-conjugated compounds.

Beyond its role as a vitamin, biotin has gained attention in drug delivery systems aimed at improving cellular uptake and drug efficacy. For example, biotin-conjugated compounds like CPT-PEG-Biotin utilize SMVT-mediated transport to enhance drug delivery efficiency in ovarian cancer cells [159]. Similarly, biotin-linked nanocarriers have shown potential in targeting acidic tumor environments, offering improved chemotherapeutic delivery while minimizing side effects and enhancing therapeutic outcomes.

### Vitamin B_9_

Vitamin B_9_, also known as folate or folic acid (FA), is a crucial coenzyme in one-carbon transfer reactions essential for DNA synthesis, cell division, and red blood cell formation. It may also play a role in biological processes related to tumorigenesis. Folate is primarily obtained from dietary sources, including liver and leafy green vegetables. Despite its availability, folate deficiency remains prevalent in developing countries and continues to be a significant public health concern in developed nations, where even mild deficiencies are relatively common [166]. These deficiencies have been linked to various neurological disorders in children, such as hereditary folate malabsorption [167], cerebral folate deficiency [168], and autism spectrum disorders [169]. In response, countries like the United States have implemented public health initiatives, such as mandatory FA fortification of grain products, to reduce the incidence of neural tube defects in newborns [170].

#### Absorption and transport of vitamin B_9_

Most dietary folate exists as a polyglutamate chain, which must be hydrolyzed to monoglutamate before absorption [171]. Folate absorption primarily occurs in the small intestine via three distinct transmembrane uptake systems [169, 172]. The proton-coupled folate transporter (PCFT), encoded by *SLC46A1*, plays a central role in absorbing dietary folate in the proximal jejunum and duodenum, where it operates optimally at acidic pH levels [167, 173]. In contrast, the RFC, as discussed in section “Absorption and transport of vitamin B_1_”, functions at a neutral pH and is the primary route for folate transport into systemic tissues [172, 174, 175]. The folate receptors (FRs), especially FRα encoded by the *FOLR1* gene, are predominantly expressed on tumor cell surfaces and minimally on normal cells [168]. The binding of folate to FR activates the Janus kinase (JAK)/signal transducer and activator of transcription 3 (STAT3) signaling pathway, which in turn activates STAT3 and regulates genes involved in cell growth and survival [176]. Additionally, multi-drug resistance-associated proteins (MRPs) and breast cancer resistance protein (BCRP), located on the apical membrane of the small intestine, contribute to folate transport by facilitating its export into the intestinal lumen [177]. Meanwhile, basolaterally localized MRP3 regulates folate absorption across enterocytes [178].

#### Distribution and metabolism of vitamin B_9_

After absorption, folate is reduced sequentially to dihydrofolate (DHF) and tetrahydrofolate (THF) by dihydrofolate reductase (DHFR), with NADPH supplying the necessary electrons for the reduction process [179]. Folate-dependent one-carbon metabolism comprises two main pathways: the folate cycle and the methionine cycle, which function in parallel and complement each other within the cytoplasm and mitochondria [180, 181]. THF is transported into the mitochondria via SLC25A32 [179, 182, 183]. The folate cycle begins with the catalysis of serine by serine hydroxymethyltransferase (SHMT), converting serine to glycine and producing 5,10-methylenetetrahydrofolate (5,10-MTHF) to support *de novo* purine and thymidylate synthesis [184]. 5,10-MTHF is then reduced to 5-methyltetrahydrofolate (5-MTHF) by methylenetetrahydrofolate reductase (MTHFR), which requires vitamin B_2_ as a coenzyme. In the methionine cycle, 5-MTHF donates a methyl group to convert homocysteine into methionine, a reaction catalyzed by methionine synthase (MTR) and dependent on vitamin B_12_ as a coenzyme [185]. The coordinated action of the folate and methionine cycles ensures the efficient transfer and utilization of one-carbon units within the cell.

#### Impact of genetic mutations of vitamin B_9_ transport protein

Genetic mutations in folate transport proteins significantly disrupt folate homeostasis, leading to severe clinical consequences. For example, mutations in *SLC46A1* cause hereditary folate malabsorption, characterized by profound folate deficiency, anemia, and neurological disorders [167]. Similarly, loss-of-function mutations in *FOLR1* result in cerebral folate deficiency, which is linked to neurodevelopmental impairments [169]. In contrast, mutations in the widely expressed *SLC19A1* gene are predominantly associated with megaloblastic anemia. These folate transporters are also critical for the absorption and efficacy of antifolate chemotherapeutic agents, including methotrexate (MTX), pemetrexed (PMX), pralatrexate (PTX), and raltitrexed (RTX). These agents target DHFR and thymidylate synthase (TS), respectively [186, 187]. Therefore, mutations in these transporters may contribute to antifolate resistance, making them an important focus of clinical research.

#### Recent advances in the transport of vitamin B_9_

Recent studies have highlighted the dual role of folate transport proteins in maintaining metabolic balance and advancing precision oncology [188]. These proteins are essential in the small intestine and kidneys, where they facilitate the efficient absorption of dietary folate and support systemic folate homeostasis. As a result, they have become a focal point for gastroenterologists, hematologists, and epidemiologists to explore the potential health impacts of folate metabolism imbalances. Concurrently, oncologists and pharmacologists are investigating the involvement of folate transport proteins in the transport of antifolate drugs, with the aim of developing innovative, personalized therapies that target these proteins.

Advancements in Cryo-EM have significantly enhanced our understanding of the structure and function of folate transport proteins [189–192]. For instance, recent studies on the RFC have revealed that TPP, not TMP as previously assumed, is the primary coupled substrate during folate transport. RFC plays a key role in maintaining the intracellular and extracellular folate balance by exchanging extracellular 5-MTHF with intracellular TPP at the same binding site [192].

Folate transport proteins have also shown substantial clinical potential in cancer therapy. PCFT, in particular, has emerged as a crucial target due to its high expression in malignant cells and its heightened activity under acidic conditions. This has led to the development of 6-substituted pyrrolo[2,3-*d*]pyrimidine antifolates [193], which exert potent antitumor effects by inhibiting glycinamide ribonucleotide formyltransferase (GARFTase), an enzyme essential for purine biosynthesis [194]. Additionally, the overexpression of FRα in tumor cells promotes effective drug delivery. For example, AGF94, which depends on both PCFT and FRα for transport, significantly enhances intracellular drug concentrations in cancer cells [193, 194]. However, the overlapping and redundant roles of PCFT, FRα, and RFC in drug transport can pose challenges to therapeutic efficacy, underscoring the need for further research into their coordinated function in antifolate-based treatments.

### Vitamin B_12_

Vitamin B_12_, also known as cobalamin or cyanocobalamin (Cbl), is crucial for various metabolic processes, including DNA synthesis, red blood cell formation, and neurological function. Although the body requires only small amounts of vitamin B_12_—since it is used by just two enzymes and is efficiently stored and recycled—deficiency remains common. Approximately 10% of Americans have low vitamin B_12_ levels, with subclinical deficiencies affecting 2.5% to 26% of the general population [195]. In developing countries, where malnutrition is more prevalent, deficiency rates are notably higher [196]. The primary sources of vitamin B_12_ are animal-based foods, such as meat, fish, dairy products, and eggs. Vegans, especially those following strict plant-based diets, are at a higher risk of vitamin B_12_ deficiency. Similarly, the elderly and individuals with conditions like gastrointestinal disorders or malabsorption syndromes are also particularly susceptible to deficiencies.

#### Absorption and transport of vitamin B_12_

Vitamin B_12_ absorption follows a complex pathway involving three key binding proteins: haptocorrin (HC), intrinsic factor (IF), and transcobalamin (TC) [197]. Initially, Cbl is released from food proteins in the stomach and binds to HC, which is secreted by the salivary glands and stomach lining. This complex protects Cbl from degradation by gastric acid [198]. As the Cbl-HC complex enters the duodenum, HC is degraded by pancreatic proteases, enabling Cbl to bind to IF. The Cbl-IF complex then travels to the ileum, where it binds to the cubam receptor on the apical surface of enterocytes. The cubam receptor, composed of cubilin and amnionless, facilitates the endocytosis of the Cbl-IF complex into the enterocytes [199–202].

Within the enterocytes, IF is degraded in lysosomes, releasing Cbl. The free Cbl is then transported across the basolateral membrane to the bloodstream via the ABC transporter protein MRP1 [203]. In the bloodstream, Cbl binds to TC, forming the TC-Cbl complex, which is delivered to tissues throughout the body to support various metabolic functions.

#### Distribution and metabolism of vitamin B_12_

The liver serves as the primary storage site for vitamin B_12_, with a portion excreted in bile and reabsorbed through enterohepatic circulation, minimizing daily losses (~0.15% of total body stores). In the plasma, approximately 80% of Cbl binds to HC, while only the TC-Cbl complex can be recognized by cellular receptors, such as transcobalamin (TC, TCII) receptor (TCblR/CD320), facilitating tissue uptake [204].

Inside cells, Cbl is transported from lysosomes to the cytoplasm by LMBD1 and ABCD4 [205, 206]. In the cytoplasm, Cbl is converted into two active coenzyme forms: methylcobalamin (MeCbl) and adenosylcobalamin (AdoCbl) [207]. MeCbl acts as a coenzyme for MTR, enabling the conversion of homocysteine to methionine and supporting DNA synthesis. AdoCbl functions in the mitochondria as a coenzyme for methylmalonyl-CoA mutase, catalyzing the conversion of methylmalonyl-CoA to succinyl-CoA. This process is vital for the metabolism of branched-chain amino acids and odd-chain fatty acids.

#### Impact of genetic mutations of vitamin B_12_ transport protein

The absorption, transport, and metabolism of vitamin B_12_ depend on the coordinated actions of several proteins, and mutations in these pathways can lead to severe metabolic disorders [195, 208]. For example, mutations in the *CD320* gene impair cellular uptake of the TC-Cbl complex, resulting in methylmalonic aciduria and homocystinuria, conditions marked by megaloblastic anemia, developmental delays, and neurological damage [209]. Mutations in the transcobalamin II (*TCN2*) gene disrupt vitamin B_12_ transport from the bloodstream to cells, causing severe anemia and neurological dysfunction, typically manifesting in infancy [208, 210, 211]. Mutations in genes of the Cubam complex (e.g. cubilin or amnionless) lead to Imerslund-Gräsbeck syndrome, a rare childhood-onset disorder of vitamin B_12_ deficiency [210, 212]. Furthermore, mutations in genes related to lysosomal and mitochondrial proteins (e.g. LMBD1, ABCD4, and MMACHC) also impair B_12_ metabolism [205]. Despite the different causes, these disorders share common symptoms, such as anemia, neurological damage, and metabolic dysfunction. Early diagnosis through genetic testing, coupled with high-dose vitamin B_12_ supplementation, is essential for improving patient outcomes.

#### Recent advances in the transport of vitamin B_12_

Recent research has identified additional proteins critical to vitamin B_12_ transport, thereby advancing our understanding of its absorption and intracellular dynamics. However, approximately 15% to 20% of hereditary cases of vitamin B_12_ malabsorption remain unexplained, suggesting that other yet-to-be-identified regulatory proteins play a role in vitamin B_12_ transport [213]. For example, the mechanism by which Cbl is transported to the mitochondria remains unclear, representing a significant gap in our knowledge. Additionally, the transport of vitamin B_12_ within the central nervous system (CNS) remains largely unexplored [197].

The discovery of new vitamin B_12_ transport proteins has important clinical implications, particularly for genetic testing and the detection of B_12_-related deficiencies. Advanced diagnostic tools, such as holo-transcobalamin measurement, improve the accuracy of vitamin B_12_ status assessments. Maintaining optimal vitamin B_12_ levels is crucial during infancy for neurodevelopment and in older adults to preserve cognitive health. These factors underscore the need for continued research into the mechanisms of vitamin B_12_ transport and the development of targeted therapies.

### Vitamin C

Vitamin C, or L-ascorbic acid (ASC), exists in the body in two primary forms: the reduced form (ASC) and the oxidized form (dehydroascorbic acid, DHA), which interconvert to maintain the total bioavailable vitamin C pool [214, 215]. As a vital antioxidant, vitamin C neutralizes free radicals by donating electrons, thereby protecting DNA and intracellular proteins from oxidative damage [216–218]. Additionally, it functions as an essential cofactor in 15 enzymatic reactions [219], most notably in collagen synthesis, where it facilitates the hydroxylation of proline and lysine. This process is crucial for maintaining the structural integrity of blood vessels, skin, muscles, and bones [220]. Since humans lack the terminal enzyme needed for endogenous vitamin C synthesis [221], dietary intake is essential. Major sources include citrus fruits and leafy green vegetables. A deficiency in vitamin C leads to scurvy, a condition marked by bleeding gums, weakened connective tissues, and impaired wound healing [222, 223]. These effects underscore the nutrient’s critical role in health, particularly for populations with limited access to fresh produce.

#### Absorption and transport of vitamin C

At physiological pH, vitamin C primarily exists as the monovalent anion ASC, which neutralizes free radicals by donating electrons. Upon donating two electrons, ASC is converted into DHA [216, 224]. Vitamin C transport occurs through passive diffusion, facilitated diffusion, and active transport mechanisms [225]. Notably, ASC is actively transported via sodium-dependent vitamin C transporters, SVCT1 (encoded by *SLC23A1*) and SVCT2 (encoded by *SLC23A2*) [215, 218, 226, 227]. SVCT1, a high-capacity transporter, is predominantly expressed in the epithelial cells of the intestine, liver, and kidneys, where it regulates gastrointestinal absorption and renal reabsorption to maintain systemic vitamin C levels [223, 228, 229]. In contrast, SVCT2 is a high-affinity transporter expressed in a variety of tissues, including the brain [218, 223, 230–232], and plays a key role in transporting vitamin C into cells during oxidative stress. Furthermore, DHA is transported by glucose transporters (GLUTs), members of the solute carrier 2A (SLC2A) family, including GLUT1, 2, 3, 4, and 8 [218, 233, 234]. Inside cells, DHA is rapidly reduced back to ASC by an enzymatic system, and this rapid intracellular recycling promotes efficient diffusion of DHA through GLUTs [215, 218].

#### Distribution and metabolism of vitamin C

Vitamin C is widely distributed throughout the body, with its concentration varying significantly across tissues based on their specific functions. The highest concentrations, up to 10 mmol/L, are found in the adrenal glands, pituitary gland, and retina, likely due to their roles in stress response and vision [235]. The brain also maintains relatively high vitamin C levels, around 5−10 mmol/L, where it helps neutralize free radicals and supports neurotransmitter synthesis. In contrast, the muscle and heart tissues have lower concentrations, approximately 0.2 mmol/L, reflecting their reduced dependence on vitamin C.

In cancer cells, vitamin C transport and utilization differ from that in normal cells. Cancer cells often overexpress the SVCT2 protein, enhancing their ability to take up vitamin C efficiently [236, 237]. In breast cancer cells, SVCT2 is primarily localized to the mitochondrial membrane [238, 239]. Cancer cells can also transport DHA through GLUTs, rapidly converting it back to ASC to reduce oxidative stress within the cells [240]. At high doses, however, vitamin C increases reactive oxygen species (ROS) levels, inducing oxidative stress that can kill cancer cells and inhibit their proliferation and cell cycle progression [240].

#### Impact of genetic mutations of vitamin C transport protein

Mutations in vitamin C transporter genes can disrupt homeostasis, leading to varying degrees of vitamin C deficiency and increased disease risk [223, 241, 242]. For instance, polymorphisms in the *SLC23A1* gene can impair intestinal vitamin C absorption, resulting in lower plasma levels and a heightened risk of conditions, such as scurvy, cardiovascular diseases, and certain cancers [224, 241]. Similarly, individuals with compromised SVCT2 function face greater susceptibility to oxidative stress and inflammatory diseases [243]. Investigating the genetic basis of vitamin C transport not only advances personalized nutrition strategies but also paves the way for precision medicine approaches in disease prevention and treatment.

#### Recent advances in the transport of vitamin C

Although SVCT1 and SVCT2 are crucial for the active transport of vitamin C, their precise roles in metabolism, especially in humans, remain poorly understood. The presence of mitochondrial SVCT2, found exclusively in cancer cells, suggests that mitochondrial vitamin C may play a vital role in cancer cell survival and development. Structural studies of different conformational states have offered valuable insights into the transport mechanisms of these proteins [244, 245]. However, the function of SVCT3 remains unclear [215], and its contribution to vitamin C transport and physiological relevance requires further exploration.

Clinically, intravenous high-dose vitamin C bypasses intestinal absorption mechanisms, significantly elevating plasma vitamin C levels and presenting a promising strategy for cancer treatment. High-dose vitamin C has been shown to act as a prodrug, delivering hydrogen peroxide (H_2_O_2_) to cancer cells, which induces cell death [238, 240]. This has reignited interest in the potential of vitamin C in cancer therapy. Despite ongoing debates regarding its efficacy, regulating plasma vitamin C levels and modulating SVCT function could become crucial strategies in developing novel cancer treatments.

**Figure 1:**
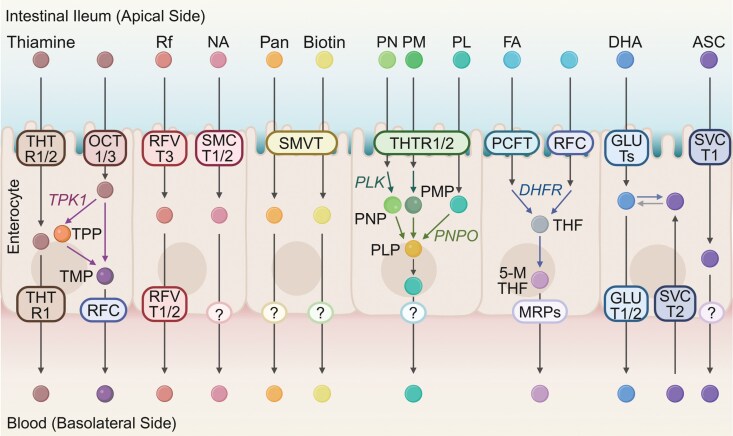
Mechanisms of water-soluble vitamin absorption in intestinal epithelial cells. Water-soluble vitamins are absorbed via specific transporters on the apical membrane and enter the bloodstream directly. Vitamin B_1_ (thiamine) is absorbed through THTR1/2, phosphorylated intracellularly to TPP by TPK1, and exported as thiamine via THTR1 or as TMP via RFC. Vitamin B_2_ (Rf) is absorbed via RFVT3 and exported by RFVT1/2. Vitamin B_3_ (NA) is absorbed through SMCT1/2. Vitamin B_5_ (Pan) and vitamin B_7_ (biotin) are absorbed by SMVT. Vitamin B_6_ (PN, PM, and PL) is absorbed through THTR1/2, phosphorylated to PNP, PMP, or PLP by PLK, with PNP or PMP further converted to PLP via PNPO. Vitamin B_9_ (FA) is absorbed by PCFT or RFC, converted to THF by DHFR and further metabolized to 5-MTHF. Vitamin C (ASC) is absorbed via SVCT1/2, while its oxidized form (DHA) enters via GLUTs. Figure created using BioRender.

The absorption of water-soluble vitamins, including vitamin C and B-vitamins, in the small intestine involves specific transport mechanisms (Fig. 1). These processes are essential for the bioavailability of these vitamins, which depend on distinct transporter systems within intestinal epithelial cells.

### Vitamin A

Vitamin A is crucial for numerous physiological functions, including vision, cellular communication, and the maintenance of skin and hair health [246, 247]. This term encompasses a group of compounds with retinol (ROL) activity, including ROL, retinal (RAL), retinoic acid (RA), and provitamin A carotenoids such as β-carotene (βC) [248]. The active metabolite RA plays a pivotal role in regulating gene expression by binding to nuclear retinoic acid receptors (RARs) and retinoid X receptors (RXRs). This interaction influences essential biological processes, including embryonic development, cell differentiation, and immune function [249]. Preformed vitamin A (ROL and retinyl esters, REs) is exclusively found in animal-derived foods such as dairy, liver, and fish. In contrast, provitamin A carotenoids are abundant in plant-based foods, including fruits, vegetables, and oils. Although vitamin A provides significant health benefits, excessive intake can cause toxicity, leading to symptoms such as headaches, nausea, blurred vision, and dry skin [250, 251]. On the other hand, vitamin A deficiency, particularly prevalent in developing countries, can result in childhood blindness and increased vulnerability to infectious diseases. These deficiencies often stem from inadequate dietary intake, malabsorption, or chronic illnesses [252, 253].

#### Absorption and transport of vitamin A

Dietary REs must be hydrolyzed to ROL before absorption. This hydrolysis occurs in the intestinal lumen through pancreatic enzymes or at the brush border membrane via retinyl ester hydrolases (REHs) [254]. Free ROL is then efficiently absorbed by enterocytes in the proximal small intestine. Inside the enterocytes, ROL binds to cellular retinol-binding protein 2 (CRBP2), stabilizing the molecule and making its uptake irreversible [255–257]. Simultaneously, βC is transported into enterocytes through membrane-associated transporters, including scavenger receptor class B type I (SR-BI), CD36, and Niemann-Pick C1-like 1 (NPC1L1) [258–260]. Once internalized, βC is enzymatically cleaved by β-carotene 15,15’-monooxygenase 1 (BCO1), producing two molecules of RAL. These RAL molecules bind to CRBP2 and are reduced to ROL by retinal reductases [261]. ROL is then re-esterified into REs through the action of lecithin retinol acyltransferase (LRAT) or acyl-CoA retinol acyltransferase (ARAT). The REs are subsequently incorporated into chylomicrons for systemic transport [262]. These RE-enriched chylomicrons enter the lymphatic system and circulate through the bloodstream. Approximately 70% of the REs are eventually absorbed by the liver, where they are stored for future use.

#### Distribution and metabolism of vitamin A

In hepatocytes, REs are hydrolyzed to ROL. ROL can either be re-esterified by LRAT for storage in hepatic stellate cells or released into the circulation [263, 264]. In the bloodstream, ROL binds to retinol-binding protein 4 (RBP4), forming a holo-RBP4 complex. This complex interacts with transthyretin (TTR), which stabilizes it and prevents renal filtration [265, 266].

The holo-RBP4 complex delivers ROL to target tissues, such as the retinal pigment epithelium (RPE). There, the membrane receptor Stimulated by Retinoic Acid 6 (STRA6) transports holo-RBP4 for processing in the visual cycle [267–269]. In the RPE, STRA6 transfers ROL to cellular retinol-binding protein I (CRBPI), where it is ultimately converted into 11-*cis*-retinal, a key component of visual photopigment synthesis [270, 271]. STRA6 is highly expressed in the RPE but absent in adult intestines and hepatocytes [272]. In contrast, the membrane transporter RBP4 receptor-2 (RBPR2) facilitates the uptake of holo-RBP4 into intestinal and hepatic cells, supporting systemic vitamin A metabolism [267, 273].

#### Impact of genetic mutations of vitamin A transport protein

Studies have demonstrated that RA regulates vitamin A transport by activating STRA6 and SR-BI [274]. RA stimulates STRA6 to bind holo-RBP4 complex, which triggers C-terminal tyrosine phosphorylation and activates downstream JAK2-STAT3/5 signaling pathways [275]. Additionally, STRA6 is involved in p53-mediated apoptosis, particularly in response to DNA damage or elevated ROS [276]. Mutations in *STRA6* disrupt these processes, leading to vitamin A imbalance and severe conditions such as Matthew-Wood syndrome. This syndrome is characterized by ocular defects, pulmonary hypoplasia, cardiac malformations, and diaphragmatic hernias [277–279].

#### Recent advances in the transport of vitamin A

Recent advances in understanding the molecular mechanisms of vitamin A transport have revealed new therapeutic opportunities. Elevated serum levels of RBP4 have been associated with cardiovascular diseases, diabetes, and obesity, driving the development of RBP4-targeting drugs [280]. Fenretinide, a synthetic retinoid, reduces serum RBP4 levels; however, it also inhibits the conversion of βC, which could impair vision [281–283]. Moreover, the role of RBPR2 in vitamin A absorption remains unclear, with ongoing debates about its localization (whether it is on the apical or basal membrane of intestinal cells) and its efficiency in absorbing holo-RBP4 complex, necessitating further investigation [284]. Additionally, the apical membrane protein responsible for ROL uptake remains unidentified, highlighting a critical gap in our understanding of vitamin A absorption.

### Vitamin D

Vitamin D, a hormone essential for maintaining calcium and phosphorus homeostasis, supports optimal bone health. It also plays a significant role in inhibiting cancer progression, preventing autoimmune diseases, and benefiting cardiovascular, dermatological, and immune systems. Known as the “sunshine vitamin”, vitamin D is synthesized in the skin through sun exposure, while dietary sources, such as fish liver, butter, and fortified dairy products, contribute minimally to its requirements, making adequate sun exposure crucial [285]. Vitamin D exists primarily in two forms: vitamin D_2_ (ergocalciferol) and vitamin D_3_ (cholecalciferol).

Lifestyle changes, geographical factors (such as living at high latitudes or spending most of the time indoors), and darker skin pigmentation have contributed to the global rise in vitamin D deficiency [286–288]. This deficiency is linked to bone disorders, including rickets and osteomalacia, as well as muscle weakness, cardiovascular diseases, autoimmune disorders, metabolic syndrome, diabetes, and increased susceptibility to infections, including severe COVID-19 [286]. With nearly 40% of adults worldwide experiencing insufficient levels of vitamin D, addressing this deficiency has become a public health priority.

#### Absorption and transport of vitamin D

Vitamin D_3_ is primarily synthesized in the skin from 7-dehydrocholesterol under UV radiation, converting into previtamin D_3_, which then undergoes thermal isomerization to form vitamin D_3_ [289]. This process is influenced by several factors, including skin exposure, UV intensity, latitude, and skin pigmentation. In contrast, vitamin D_2_ is derived from plants and fortified foods, synthesized through UV radiation of plant sterols.

Dietary vitamin D is emulsified by bile and enters the small intestine as part of mixed micelles. Specific transporters, such as SR-BI, CD36, and NPC1L1 [290], facilitate its absorption in intestinal epithelial cells. The efflux of vitamin D in the intestine depends on ABC transporters, including ABCB1 (P-glycoprotein/MDR1) and ABCG5/ABCG8. Once absorbed, vitamin D follows lipid transport pathways, entering the lymphatic system and eventually being distributed throughout the body via the bloodstream. Adipose tissue has been identified as the primary storage site for vitamin D.

#### Distribution and metabolism of vitamin D

Vitamin D undergoes two key hydroxylation steps to become biologically active. First, vitamin D-binding protein (DBP) transports vitamin D to the liver, where it is converted by the enzyme cytochrome P450 family 2 subfamily R member 1 (CYP2R1) into 25-hydroxyvitamin D_3_ [25(OH)D_3_], also known as calcidiol [291, 292]. This circulating form serves as the primary biomarker for vitamin D status. In the kidneys, 25(OH)D_3_ undergoes further hydroxylation by cytochrome P450 family 27 subfamily B member 1 (CYP27B1, 25-hydroxyvitamin D_3_ 1-alpha-hydroxylase) to produce the hormonally active 1α,25-dihydroxyvitamin D [1α,25(OH)_2_D_3_] [293].

Active 1α,25(OH)_2_D_3_ exerts its effects by binding to the vitamin D receptor (VDR), which forms a heterodimer with the RXR [294]. The VDR-RXR complex binds to vitamin D response elements (VDREs) in the promoter regions of target genes, regulating their expression. Excess 25(OH)D_3_ and 1α,25(OH)_2_D_3_ are catabolized by cytochrome P450 family 24 subfamily A member 1 (CYP24A1), which hydroxylates them into 24-hydroxylated metabolites, initiating their inactivation [295].

#### Impact of genetic mutations of vitamin D transport protein

DBP, encoded by the group-specific component (*GC*) gene, is crucial for transporting vitamin D metabolites and maintaining their bioavailability in the bloodstream. Genetic variations, including mutations or polymorphisms in the *GC* gene, can alter DBP levels or function. For example, certain polymorphisms reduce DBP’s binding efficiency, impairing vitamin D transport and increasing the risk of diseases such as type 2 diabetes [296]. Rare pathogenic variants can cause severe DBP deficiency, leading to substantial vitamin D deficiency [297]. Genome-wide association studies have identified loci, such as *SH2B3* and *GSDMA*, that influence DBP levels. Elevated DBP levels correlate with higher 25(OH)D_3_ concentrations and a lower risk of diseases like multiple sclerosis and rheumatoid arthritis [298]. These findings highlight the critical role of DBP in vitamin D homeostasis and its broader health implications.

#### Recent advances in the transport of vitamin D

Recent research has expanded our understanding of vitamin D transport, absorption, and metabolism. While proteins such as SR-BI and NPC1L1 mediate vitamin D absorption, the specific transporter for 25(OH)D_3_ remains unidentified. Notably, gender differences in vitamin D accumulation observed in *ABCG5/ABCG8*-deficient mice suggest that sex-specific factors may influence vitamin D transport and metabolism. The VDR, expressed in nearly all human tissues, regulates over 100 proteins, including those involved in energy metabolism and immune function. In adipose tissue, VDR modulates fat storage, energy metabolism, and inflammation. In macrophages, VDR enhances innate immunity by promoting antimicrobial peptides, such as cathelicidins and defensins. These functions underscore the potential of vitamin D in immune modulation, cancer prevention, and the treatment of chronic inflammation. Additionally, VDR upregulates the expression of key transporters like the solute carrier organic anion transporter family member 1A2 (SLCO1A2) and solute carrier family 30 member 10 (SLC30A10) [299, 300], which facilitate the absorption of trace elements, including zinc, manganese, and iron. This suggests promising avenues for improving drug delivery systems. Future research should prioritize identifying specific vitamin D transporters, exploring sex-specific differences in transport and metabolism, and developing non-hypercalcemic VDR modulators. These efforts could enhance therapeutic outcomes without elevating calcium levels.

### Vitamin E

Vitamin E plays a critical role in protecting cells from damage caused by reactive oxygen and nitrogen species (ROS/RNS) and enhancing the activity of antioxidant enzymes [301–303]. It also regulates gene expression, supports cell growth, and modulates inflammation and immune function [302, 304]. Vitamin E consists of eight fat-soluble isoforms: tocopherols (α, β, γ, and δ) and tocotrienols (α, β, γ, and δ), with α-tocopherol being the most biologically active isoform due to its superior bioavailability [305, 306]. The primary dietary sources of vitamin E include vegetable oils, seeds (e.g. sunflower seeds and almonds), and various fruits and vegetables. However, global vitamin E consumption remains insufficient, with 82% of individuals failing to meet the recommended daily intake of 15 mg [307]. Deficiencies in vitamin E can result in mild hemolytic anemia and non-specific neurologic deficits [308].

#### Absorption and transport of vitamin E

Dietary vitamin E, primarily in ester form, undergoes hydrolysis by cholesterol esterase to its free form. It is then solubilized into mixed micelles by bile salts, facilitating its absorption [305, 309]. These micelles are transported into intestinal epithelial cells via passive diffusion or receptor-mediated pathways involving SR-BI, NPC1L1, and CD36 [310–312]. Once inside the enterocytes, vitamin E combines with triglycerides to form chylomicrons (VitE-CM). These chylomicrons are transported into the lymphatic system with the assistance of ABCA1, ABCG5/ABCG8, and apolipoproteins A-containing lipoproteins (ApoA-I), which help form nascent high-density lipoprotein (HDL) particles [313–316]. Following lymphatic transport, VitE-CM enters the bloodstream via the thoracic duct. In circulation, vitamin E binds non-specifically to plasma lipoproteins, such as HDL and LDL. Lipoprotein lipase (LPL) hydrolyzes the triglycerides in chylomicrons, leaving behind remnants for further transport [305, 317].

#### Distribution and metabolism of vitamin E

The liver plays a central role in distributing vitamin E. The α-tocopherol transfer protein (α-TTP) selectively transports α-tocopherol to plasma lipoproteins (e.g. VLDL, HDL, and LDL), maintaining stable concentrations in blood and tissues [315, 318]. Other forms, such as γ-tocopherol, are primarily metabolized in the liver. This explains why α-tocopherol comprises 90% of plasma vitamin E, despite γ-tocopherol accounting for 70% of dietary intake [319]. Phospholipid transfer protein (PLTP) facilitates the transfer of α-tocopherol between lipoproteins like HDL and LDL, promoting its dynamic redistribution in the plasma [320]. PLTP also participates in VLDL assembly and secretion, contributing to the hepatic biogenesis of ApoB-I [308, 321]. Additionally, tocopherol-associated proteins (TAPs) assist in intracellular vitamin E transport. For example, TAP1 is thought to transport α-tocopherol to the mitochondria, especially under certain pathological conditions [315, 322, 323].

Vitamin E metabolism occurs in three phases, akin to other xenobiotics [324]. In Phase I, cytochrome P450 enzymes (e.g. CYP3A4 and CYP4F2) hydroxylate vitamin E, producing long-chain metabolites that undergo further breakdown by β-oxidation into short-chain carboxyethyl hydroxychromans (CEHCs) [325–327]. During Phase II, these CEHCs conjugate with glucuronic acid or sulfate to increase their water solubility. In Phase III, the conjugated metabolites are excreted via bile or urine through transporters, such as multi-drug resistance proteins (MDRs) and MRPs [328].

#### Impact of genetic mutations of vitamin E transport protein

Mutations or dysfunctions in vitamin E transport proteins can disrupt vitamin E status and increase the risk of deficiency-related conditions. For example, mutations in the *TTPA* gene, which encodes α-TTP, lead to ataxia with vitamin E deficiency, a neurodegenerative disorder marked by low plasma α-tocopherol levels [329]. High-dose vitamin E supplementation has been shown to slow disease progression in affected individuals. Deficiencies in PLTP significantly reduce α-tocopherol levels in the liver, brain, and peripheral tissues, leading to oxidative stress and anxiety-like behaviors, which suggest an increased risk of neurodegenerative diseases [330, 331]. In contrast, overexpression of PLTP can enhance hepatic VLDL secretion and elevate plasma ApoB levels, increasing the risk of atherosclerosis [332]. Furthermore, a single-nucleotide polymorphism in the *CD36* gene has been linked to plasma α-tocopherol concentration, which may indirectly affect vitamin E metabolism [333]. A deeper understanding of these transport proteins could enable the development of personalized therapies to improve outcomes for individuals with specific genetic mutations.

#### Recent advances in the transport of vitamin E

Recent studies have underscored the complex interactions between vitamin E transport and drug metabolism. Drugs like ezetimibe and orlistat can significantly impair vitamin E absorption by inhibiting intestinal receptors such as NPC1L1 [259, 316, 334]. Additionally, vitamin E functions as a xenobiotic, activating pregnane X receptor (PXR), which induces CYP3A enzyme expression. This accelerates the metabolism of vitamin E and may alter the effectiveness of other medications [335]. Vitamin E has also shown therapeutic promise in non-alcoholic steatohepatitis, improving liver function and histological outcomes when combined with lifestyle changes or drugs like pioglitazone [302]. While its antioxidant properties are widely acknowledged for preventing coronary heart disease and atherosclerosis, excessive supplementation may disrupt physiological ROS signaling, potentially impairing cellular function [303, 309, 336, 337]. This dual nature underscores the need for further research to refine the clinical applications of vitamin E.

### Vitamin K

Vitamin K was originally identified for its role in blood clotting, with the “K” derived from the Danish word “koagulation” [338]. Beyond this function, vitamin K plays a variety of crucial physiological roles, including antioxidation, anti-inflammation, cancer prevention, cardiovascular health promotion, and bone support [339–341]. There are two primary forms of vitamin K found in nature: vitamin K_1_ (phylloquinone, PK), the main dietary source, which is abundant in leafy green vegetables such as kale and spinach [342, 343], and vitamin K_2_ (menaquinones, MK-n), synthesized by gut microbiota and found in fermented foods like cheese, curds, and natto. MK-4, the only menaquinone not synthesized by bacteria, is present in dairy products and certain meats [344–346]. Vitamin K_3_ (menadione, MD), a synthetic form, was banned from dietary supplements due to safety concerns but is still commonly used in animal feed [347]. MD is a metabolic product of PK and serves as a precursor for MK-4 synthesis in tissues [348, 349]. Although vitamin K deficiency is rare in adults, certain high-risk groups are more susceptible. These include individuals with fat malabsorption disorders, those on long-term vitamin K antagonists (e.g. warfarin), newborns, and patients on prolonged broad-spectrum antibiotics [342, 350, 351]. Reports of systemic toxicity from natural vitamin K are virtually nonexistent [342].

#### Absorption and transport of vitamin K

Vitamin K absorption relies on mixed micelles formed by bile salts, which facilitate its entry into intestinal epithelial cells. This process is mediated by membrane transporters, including SR-BI, CD36, and NPC1L1 [352, 353]. Once inside enterocytes, PK undergoes enzymatic cleavage to remove its side chain, converting it into MD [348]. To prevent MD accumulation and potential toxicity, the ABCG5/ABCG8 transporters actively expel excess MD into the intestinal lumen [354]. The absorbed PK, menaquinones (MK-n), and MD are then transported to the ER, where they are packaged into chylomicrons (VitK-CMs) with the involvement of ApoA and ApoB-48 [355, 356]. These VitK-CMs enter systemic circulation via the lymphatic system, delivering vitamin K to various tissues [357].

#### Distribution and metabolism of vitamin K

Vitamin K distribution and metabolism vary across tissues. PK is primarily concentrated in the liver, where it supports the synthesis of coagulation factors, including II, VII, IX, and X [358]. In contrast, MK-n is distributed to extrahepatic tissues, where they serve as cofactors for the enzyme γ-carboxylase. This enzyme catalyzes the carboxylation of vitamin K-dependent proteins (VKDPs), such as osteocalcin and matrix Gla protein, which promote bone health and prevent vascular calcification [339, 359, 360]. For example, MK-4 is preferentially transported to the pancreas and brain, where it exerts anti-inflammatory and neuroprotective effects via the Gas 6 receptor [361]. MK-7 is transported to the bone and kidneys, where it regulates calcium homeostasis and supports vascular health. PK, MK-n, and MD are converted into bioactive MK-4 by UbiA prenyltransferase domain-containing protein 1 (UBIAD1) enzymes [348, 362]. Due to the body’s limited capacity to store vitamin K, the vitamin K cycle plays a crucial role in maintaining its biological activity. This cycle involves the reduction of vitamin K epoxide (KO) to its active form, reduced vitamin K hydroquinone (KH_2_), by vitamin K epoxide reductase (VKOR) and vitamin K reductase (VKR) [363]. This process ensures the recycling and sustained activity of VKDPs.

#### Impact of genetic mutations of vitamin K transport protein

Mutations in proteins responsible for vitamin K transport can result in significant metabolic and clinical disorders. For instance, mutations in the *ABCG5* and *ABCG8* genes are linked to sitosterolemia, a condition that disrupts sterol metabolism, impairs vitamin K distribution, and heightens deficiency risks. These disruptions adversely affect coagulation and bone health [354, 364].

#### Recent advances in the transport of vitamin K

Recent research on vitamin K has identified its reduced form (KH_2_) as a potent ferroptosis inhibitor. By scavenging lipid peroxides and enhancing antioxidant defenses [365, 366], KH_2_ prevents iron-dependent cell death. This breakthrough offers promising therapeutic potential for cancer treatment, as many cancer cells exhibit heightened sensitivity to ferroptosis.

In patients with chronic kidney disease, impaired HDL functionality significantly reduces MK-7 transport and cellular uptake, diminishing its effectiveness in preventing vascular calcification [367, 368]. Similarly, cholesterol absorption inhibitors such as ezetimibe may lower vitamin K absorption due to shared transport pathways. Warfarin also disrupts the vitamin K cycle, increasing the risk of osteoporosis and vascular calcification. These findings underscore the importance of tailored vitamin K supplementation strategies, alongside interventions that restore HDL functionality or regulate vitamin K metabolism in specific populations.

**Figure 2:**
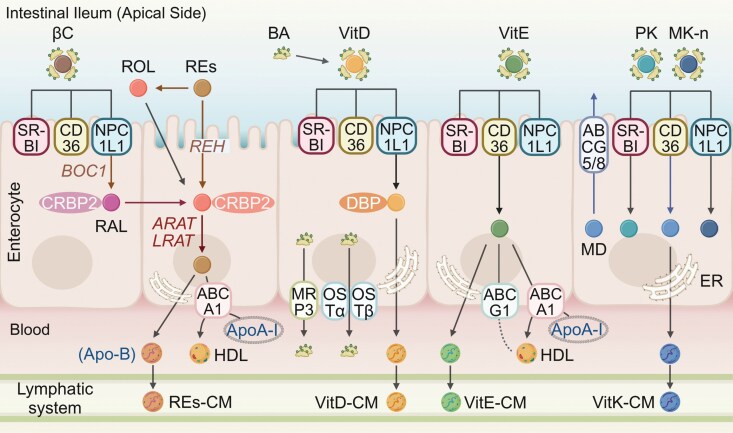
Mechanisms of fat-soluble vitamin absorption in intestinal epithelial cells. Fat-soluble vitamins are absorbed into enterocytes through broad-specificity transporters (SR-BI, CD36, and NPC1L1), metabolized, and secreted into the lymphatic system via chylomicrons. Vitamin A is absorbed as βC or REs, metabolized into RAL or ROL bound to CRBP2, converted into REs by LRAT/ARAT enzymes, then incorporated into chylomicrons for ApoB-mediated export or transported via HDL through the ABCA1/ApoA-I pathway. Vitamin D is carried by DBP. Vitamin E is transported into HDL via ABCA1. Vitamin K is absorbed as K1 (phylloquinone) or MK-n (menaquinones) via NPC1L1 and exported by ABCG5/ABCG8. Figure created using BioRender.

Unlike water-soluble vitamins, fat-soluble vitamins—including vitamins A, D, and E—require specialized absorption mechanisms involving micelles, bile salts, and specific lipid transporters. Figure 2 illustrates the intestinal epithelial absorption process for these vitamins, emphasizing the roles of chylomicrons and lipid-based transport systems.

## Future perspectives

Vitamins are vital for numerous physiological processes, including energy metabolism, nucleic acid synthesis, lipid metabolism, and antioxidant defense. Water-soluble vitamins and vitamin K primarily function as coenzymes or cofactors, enabling critical enzymatic reactions. Vitamins A and D regulate gene expression through nuclear receptors such as RAR and VDR, influencing embryonic development, tissue differentiation, calcium homeostasis, and immune function. Antioxidant vitamins (A, E, K, and C) neutralize free radicals, maintaining cellular homeostasis, with synergistic effects observed between vitamins E and C. Additionally, vitamins B_9_ and B_12_ are essential for hematopoiesis, while vitamin B_12_ also supports myelin formation and repair, safeguarding neurological function. This review explores the transport proteins involved in the ADME of water- and fat-soluble vitamins, their biological roles, and insights into associated metabolic disorders and disease mechanisms.

## Roles of SLC transporters in water-soluble vitamins

**Figure 3:**
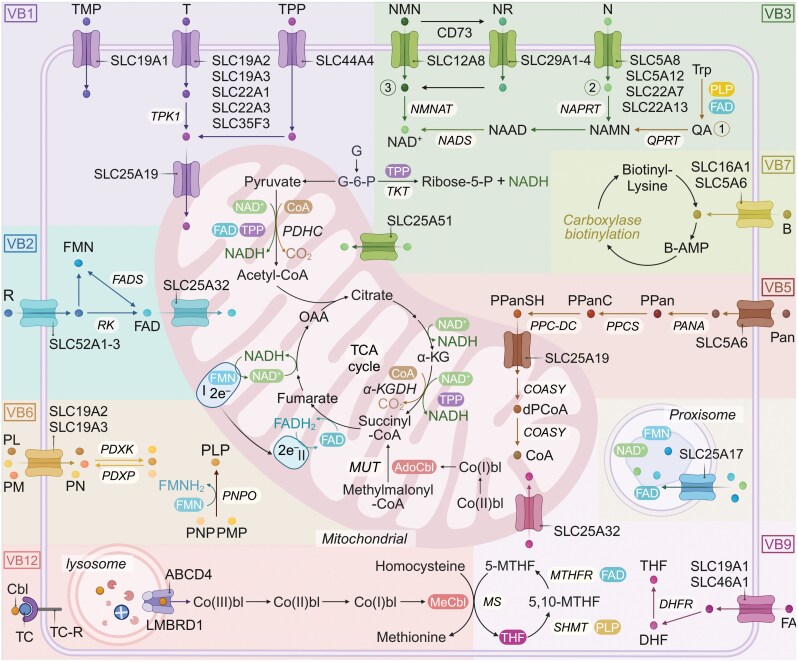
Schematic diagram showing the intracellular transport and metabolism of water-soluble vitamins. Key transporters, including the SLC family, mediate cellular uptake and distribution of water-soluble vitamins, which are enzymatically converted into coenzymes for metabolic pathways. For example, vitamin B_1_ (thiamine) is converted to TPP to support glycolysis and the pentose phosphate pathway. Vitamin B_2_ (Rf) forms FMN and FAD for redox reactions. Vitamin B_3_ (NA) generates NAD^+^/NADP^+^ for energy metabolism. Vitamin B_5_ (Pan) produces CoA for fatty acid metabolism and TCA cycle processes. Vitamin B_6_ (PL, PN, and PM) is activated as PLP for amino acid metabolism. Vitamin B_7_ (biotin) is covalently attached to carboxylases to facilitate gluconeogenesis and fatty acid metabolism. Vitamin B_9_ (FA) is involved in nucleotide synthesis, amino acid metabolism, and one-carbon metabolism. Vitamin B_12_ (Cbl) supports methyl transfer and DNA synthesis. Figure created using BioRender.

The absorption of water-soluble vitamins relies predominantly on SLC family transporters, which mediate transmembrane transport and intracellular compartmentalization. For instance, the SLC52 family is responsible for Rf transport, while the SLC23 family facilitates sodium-dependent vitamin C uptake. Intracellularly, mitochondrial transporters, such as SLC25A42, SLC25A32, and SLC25A51/52, are crucial for the transport of CoA, FAD, and NAD^+^, respectively. In the ER, SLC33A1 regulates acetyl-CoA transport, while SLC25A17 facilitates CoA translocation into peroxisomes. These highly specific mechanisms underscore the precision of vitamin compartmentalization and offer valuable insights for developing therapies to address transporter deficiencies. Figure 3 provides a detailed schematic diagram of the intracellular transport and metabolism of water-soluble vitamins, highlighting their pathways to target tissues and subsequent metabolic transformations, both of which are essential for maintaining cellular function and overall health.

## Active transport and homeostasis of fat-soluble vitamins

Recent studies have greatly advanced our understanding of fat-soluble vitamin transport, revealing a dynamic and complex system that extends beyond the previously accepted model of passive diffusion. Key proteins, such as SR-BI and CD36, located on the apical membrane of intestinal epithelial cells, are essential for the uptake of vitamins D, E, K, and carotenoids. Additionally, efflux pathways mediated by ABCB1 and ABCG5/ABCG8 help maintain systemic homeostasis by transporting these vitamins back into the intestinal lumen or circulation. After absorption in the intestine, these vitamins are distributed to target tissues with the help of plasma binding proteins, including RBP and DBP, which ensure their precise delivery. Dysregulation of these transport mechanisms is closely linked to metabolic disorders, such as insulin resistance, obesity, and type 2 diabetes. For example, disruptions in the transintestinal cholesterol excretion (TICE) pathway can lead to excessive loss of fat-soluble vitamins, exacerbating metabolic dysregulation (TIME effect) [284].

## Metabolic synergy and future directions

Water- and fat-soluble vitamins exhibit remarkable metabolic synergy. For example, FAD and FMN are crucial for vitamin D activation, while PLP regulates retinol metabolism by maintaining the redox balance of vitamin A. Homocysteine metabolism depends on the coordinated activity of vitamins B_6_, B_9_, and B_12_. Additionally, the kynurenine pathway, which synthesizes NAD^+^ from Trp, is tightly controlled by B-vitamins. Deficiencies in these vitamins disrupt these processes, leading to metabolic abnormalities. For instance, vitamin B_12_ deficiency triggers the “methylfolate trap”, impairing folate metabolism and DNA synthesis.

Despite recent advances, significant gaps in our understanding remain. For example, the specific apical membrane transporters for fat-soluble vitamins, such as vitamin A, have yet to be identified. Furthermore, the mechanisms underlying intracellular vitamin compartmentalization within organelles, including the mitochondria, ER, and peroxisomes, require further exploration to elucidate their roles in vitamin distribution and metabolic regulation. Investigating genetic variations that influence vitamin absorption will lay the groundwork for personalized nutrition strategies. Additionally, studies using disease models to examine the link between transporter dysfunction and metabolic disorders will support the development of precision therapies targeting metabolic diseases.
